# Standard views do not suffice in assessing distal scaphoid articular cannulated screw penetration

**DOI:** 10.1186/s10195-023-00735-1

**Published:** 2023-11-09

**Authors:** Pierre-Emmanuel Chammas, Maxime Pastor, Michel Chammas, Geert Alexander Buijze

**Affiliations:** 1 Hand and Upper Extremity Surgery Unit, CHU Lapeyronie, University of Montpellier, 371 Av. du Doyen Gaston Giraud, 34090 Montpellier, France; 2 Department of Radiology, CHU Lapeyronie, University of Montpellier, Montpellier, France; 3 Hand, Upper Limb, Peripheral Nerve, Brachial Plexus and Microsurgery Unit, Clinique Générale Annecy, Annecy, France; 4 Department of Orthopaedic Surgery, Amsterdam UMC, University of Amsterdam, Amsterdam, Netherlands

**Keywords:** Scaphoid fracture, Screw fixation, Cannulated screw, Hardware problems, Screw penetration, Arthritis

## Abstract

**Background:**

Articular screw penetration is one of the most common hardware-related problems after scaphoid fracture fixation, occurring in up to two-thirds of patients, in particular into the scaphotrapezotrapezoidal (STT) joint. The aim of this study was to investigate whether this clinically important issue could be detected using standard anteroposterior (AP) and lateral, as well as additional nonstandard fluoroscopic views using direct open visualization with magnifying loupes as reference standard.

**Materials and methods:**

Ten fresh cadaver wrists were used for this imaging study. A 2.2 mm cannulated compression screws with a length of 24 mm was placed in the scaphoid and incrementally left to protrude at the STT joint up to 2 mm. Eight fluoroscopic views of the wrist were then obtained by rotating the forearm using goniometric measurements, keeping the image beam parallel to the floor: (1) anteroposterior with the wrist in neutral rotation, (2) anteroposterior with the wrist in ulnar deviation, (3) supinated oblique 60° from neutral (60° supinated oblique), (4) supinated oblique 45° from neutral (45° supinated oblique), (5) a true lateral, (6) a true lateral with the wrist in radial deviation, (7) pronated oblique 45° from neutral (45° pronated oblique), and (8) a pronated oblique 60° from neutral (60° pronated oblique).

**Results:**

Standard anteroposterior and lateral fluoroscopy views (radiographically calibrated) of a percutaneous cannulated screw fixation of a scaphoid fracture were insufficient to detect distal articular penetration, missing half the amount of screw penetrations in the current study. The 45° pronated oblique view was found as the most sensitive in detecting STT penetration (*p* < 0.0001).

**Conclusions:**

Standard anteroposterior and lateral fluoroscopy views of a percutaneous cannulated screw fixation of a scaphoid waist fracture are insufficient to detect STT screw penetration. According to the current study, standard views would have missed half the amount of screw penetrations, which seems to reflect the high incidence of this problem in current practice. The most sensitive view was the 45° pronated oblique view, which detected STT screw penetration in all cases.

*Level of Evidence* Not applicable.

## Introduction

**Fig. 1:**
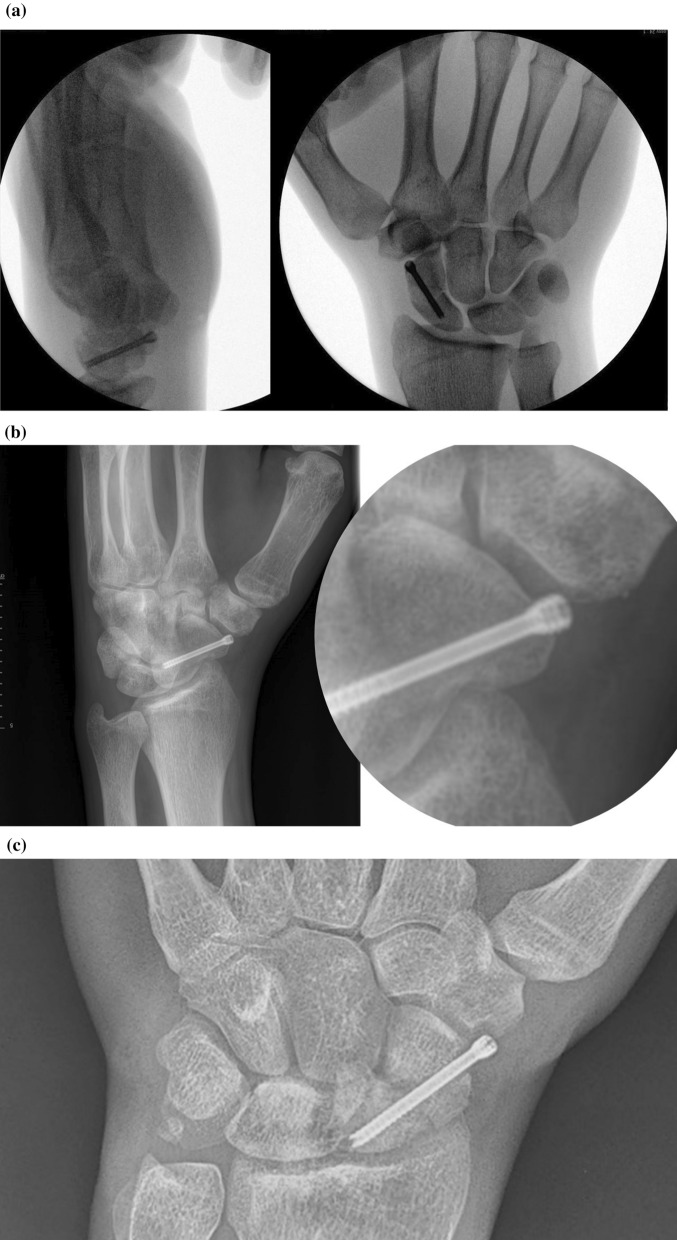
**A** Standard anteroposterior and lateral fluoroscopy views of a percutaneous cannulated screw fixation of a scaphoid waist fracture showing minimal STT screw penetration. **B** Postoperative semi-pronated radiograph showing significant distal articular screw penetration and absence of screw thread purchase in the distal fragment. **C** Another example of significant distal articular screw penetration with subsequent delayed union of a scaphoid waist fracture

Problems related to hardware are likely the most common complication of scaphoid fracture fixation and remain either underreported or underemphasized, with highly heterogeneous rates across the literature [1–3]. Likely due to an increasing trend toward percutaneous retrograde cannulated screw fixation of scaphoid fractures, articular screw penetration seems to be the most common problem, in particular at the scaphotrapeziotrapezoidal (STT) joint (Fig. 1). In the largest scaphoid fracture fixation trial to date (SWIFFT Trial), screw penetration was identified in 93 (65%) of 142 patients [2]. In a meta-analysis comparing operative versus conservative treatment for nondisplaced scaphoid waist fractures, STT osteoarthritis developed in 40% of patients after surgery as compared with 10% of patients after cast immobilization [4].

A previous cadaveric study focused only on screw penetration of the proximal scaphoid articular surface and found that the semi-pronated (60°) oblique view was the most important fluoroscopic image to obtain [5]. However, data from the SWIFFT trial showed that the distal articular surface is at higher risk, with screw penetration occurring in 60–66.7% of patients, with a mean penetration of 1.7 mm at the STT joint [6]. No study has yet evaluated which fluoroscopic view is the most sensitive for the more prevalent distal articular (STT) scaphoid screw penetration.

The aim of this study was to investigate whether this overly common and clinically important issue could be detected using standard anteroposterior (AP) and lateral, as well as additional nonstandard fluoroscopic views using direct open visualization with magnifying loupes as reference standard, to prevent distal articular screw penetration, delayed union, and symptomatic STT arthritis. As a secondary aim, the addition role of the 45° semi-pronation view (versus a previously demonstrated 60° semi-pronation view) is studied.

## Materials and methods

Ten fresh cadaver wrists were used for this imaging study. There were no visible wrist scars to be noted on any of the cadavers and no radiographic signs of STT arthritis. A mini-open incision with capsulotomy were performed at the STT joint to expose the entry point of the screw at the volar scaphoid tubercle. A 2.2 mm cannulated compression screws (Medartis SpeedTip®) with a length of 24 mm was advanced over a pre-drilled K-wire in the scaphoid along its longitudinal axis. The screw was placed under direct vision until it was flush with the subchondral bone and its position confirmed fluoroscopically with an image intensifier (OEC Elite MiniView, GE Hualun Medical Systems, Co. Ltd). Eight fluoroscopic views of the wrist were then obtained by rotating the forearm using goniometric measurements, keeping the image beam parallel to the floor: (1) anteroposterior with the wrist in neutral rotation, (2) anteroposterior with the wrist in ulnar deviation, (3) supinated oblique 60° from neutral (60° supinated oblique), (4) supinated oblique 45° from neutral (45° supinated oblique), (5) a true lateral, (6) a true lateral with the wrist in radial deviation, (7) pronated oblique 45° from neutral (45° pronated oblique), and (8) a pronated oblique 60° from neutral (60° pronated oblique).

**Fig. 2:**
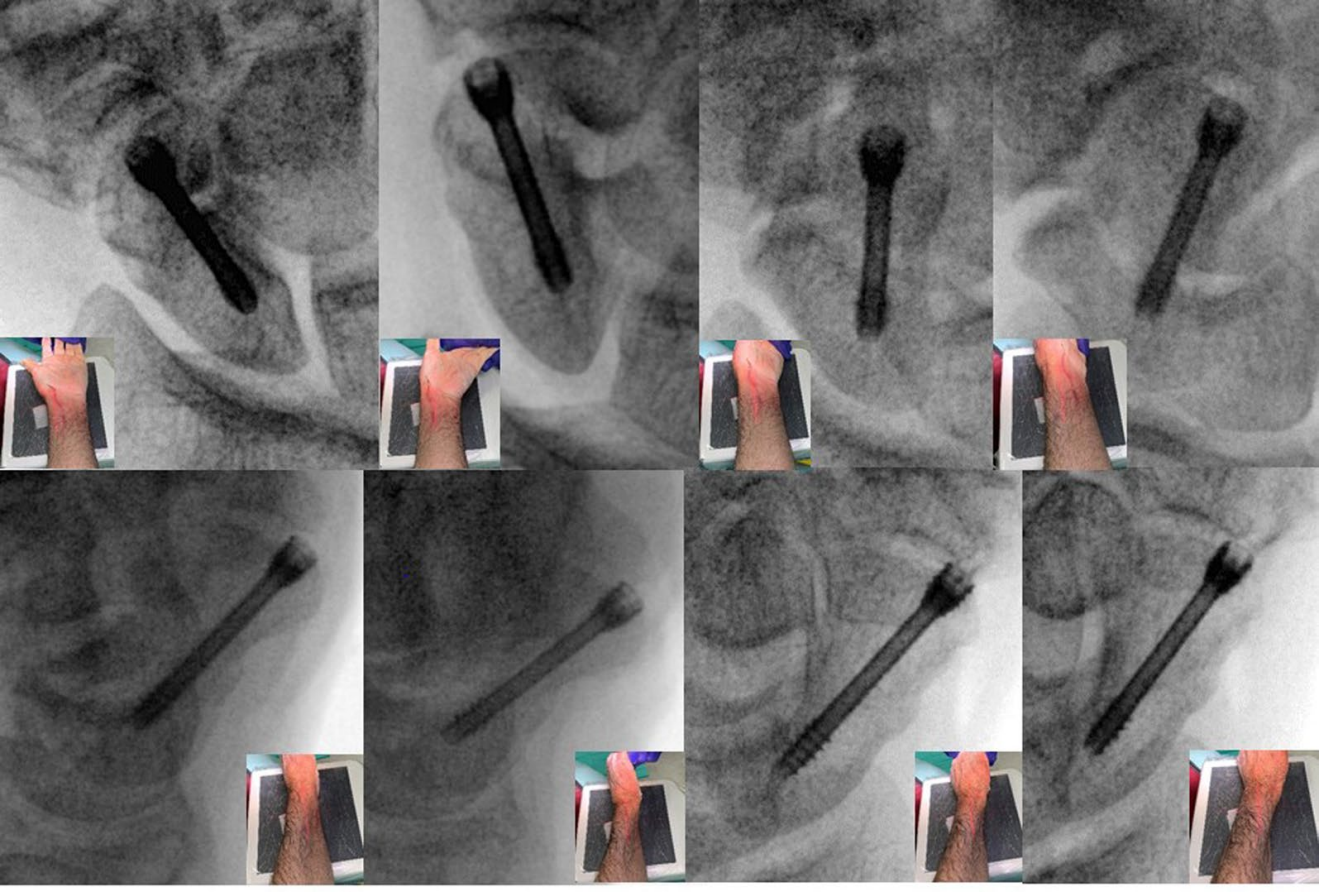
Eight fluoroscopic views: (1) anteroposterior with the wrist in neutral rotation, (2) anteroposterior with the wrist in ulnar deviation, (3) 60° supinated oblique, (4) 45° supinated oblique, (5) a true lateral, (6) a true lateral with the wrist in radial deviation, (7) 45° pronated oblique, and (8) 60° pronated oblique view

In the first part of the study—according to a published study protocol [5]—the screw was then reversed to visually protrude distally from the articular surface in 0.5 mm increments up to 2 mm, measured with a caliper from the radial side of the screw, and all views were repeated (Fig. 2). At each 0.5 mm increment, visual inspection using magnifying loupes of the STT joint was performed to assess for distal articular screw penetration, and all fluoroscopic views were evaluated to determine whether cortical protrusion could be detected. The study was completed once cortical protrusion was detected on a single fluoroscopic view.

In the second part of the study, all screws were reversed so as to allow visual protrusion distally of 2 mm, measured with a caliper from the radial side of the screw visualized with magnifying loupes, and all views were repeated (Fig. 2). Calibrated measurements were performed by an independent musculoskeletal radiologist using a metal sphere measuring 10 mm in diameter present on each fluoroscopic view (OsiriX open-source software) and the average measurements of screw penetration were recorded for each view.

A Cochran’s *Q*-test using the SPSS package for data analysis (version 26.0, IBM, Armonk, NY) was run to determine whether the direction of the radiograph (using the eight different views) had an effect on the determination of articular penetration. A Kruskal–Wallis test for nonparametric continuous variables was used for comparison of the amount of screw penetration between different fluoroscopic views. The level of statistical significance was set at the 0.05 level (two sided).

## Results

With a screw penetrating 0.5 mm at the distal articular surface, standard anteroposterior with the wrist in neutral rotation and true lateral views only identified penetration in 2 and 3 out of the 10 cadaveric wrists, respectively. The 45° pronated oblique view was found as the most sensitive in detecting distal articular penetration (*p* < 0.0001), identifying screws penetrating 0.5 mm in 8 out of 10 wrists, and in all wrists following the protocol with increments up to 1.5 mm (Table 1). The average minimal amount of screw penetration required to be detected on the most sensitive fluoroscopic view—the 45° pronated oblique view—was 0.65 ± 0.5 mm.

**Table 1:** Measurements of the eight fluoroscopic views

Fluoroscopic view	Number of penetrating	Average distance measured
	Screws detected (*n* = 10)	With 2 mm penetration (mm)
Anteroposterior with the wrist in neutral rotation	2	0.2
Anteroposterior with the wrist in ulnar deviation	1	0.2
Supinated oblique 60° from neutral	0	0
Supinated oblique 45° from neutral	0	0
True lateral	5	0.7
True lateral with the wrist in radial deviation	8	1.9
Pronated oblique 45° from neutral	10^#^	2.1^#^
Pronated oblique 60° from neutral	7	1.6

^#^Significant *p* < 0.0001

In the second part of the study, screws that were visually measured to protrude distally by 2 mm were radiographically measurable on all but both semi-supinated views (Table 1). The 45° pronated oblique view had the longest measurable screw protrusion (2.1 mm, *p* < 0.0001) followed by the true lateral view with the wrist held in radial deviation (1.9 mm) and the 60° pronated oblique view (1.6 mm).

## Discussion

Screw protrusion is likely the most common surgical complication after scaphoid fracture fixation [2]. In the SWIFFT trial, the screw penetrated joints in far more patients than anticipated: in half of cases, the screw protruded 1–2 mm into the joint and in a quarter there was significant protrusion of over 2 mm. The concern about such screw protrusion is irreversible injury to the articular cartilage, leading to early degenerative arthritis in the involved joint. In most participants, screw penetration was identified on a postoperative computed tomography (CT) scan at 1 year follow-up. These findings emphasize the need for careful imaging during surgery.

As opposed to volar (mini-)open approaches, percutaneous retrograde scaphoid screw fixation not only presents potential problems at the proximal pole [7] but more prevalently at the distal articular surface with consequent symptomatology and secondary surgery [2, 6]. In the SWIFFT trial, eight (4%) of 219 patients underwent reoperation to remove prominent screws. Based on postoperative CT scans of operated patients, the most common localization of screw penetration was the STT joint (60.0–66.7%), followed by the radioscaphoid joint (44.4–55.0%) and scapholunate or scaphocapitate joint (0–23.8%) [2, 6]. The maximum screw protrusion was, on average, 1.7 mm (range 0.4–4.7 mm).

Another study on screw fixation of scaphoid fractures showed that in four of eight patients with scaphotrapezial joint space narrowing, osteoarthritic changes were related to attrition from prominence of the screw [8]. However, screw prominence and the presence of lucency around it was not predictive of an increased pain score, nor were the presence of osteoarthritis in the radioscaphoid, scaphotrapezial, or scapholunate joints.

At 12 years follow-up, Saedén et al. reported an incidence of STT osteoarthritis of 61% (14 of 23) following scaphoid fracture fixation, and only 25% (4 of 16, *p* = 0.049) after nonoperative treatment and suggested this difference resulted from possible iatrogenic STT injury [9]. Six of these patients reported symptoms affecting their work or leisure activities. Five other patients complained of symptoms but showed no radiological signs of STT osteoarthritis. Consistently, radiological signs of scaphotrapezial arthritis were not related to symptoms.

According to our results, standard anteroposterior and lateral fluoroscopy views of a percutaneous cannulated screw fixation of a scaphoid fracture are insufficient to detect STT penetration. The fact that standard views missed half the amount of screw penetrations in the current study seems to reflect the high incidence of this problem in current practice [2, 6]. On standard AP and lateral fluoroscopic views, screws protruding distally by 2 mm radiographically seem to protrude less than a millimeter on average, as overprojecting scaphoid tubercle contours obscure hardware, hindering interpretation. Missing articular penetration of hardware on two perpendicular views of a three-dimensional hemisphere (such as the scaphoid tubercle) is a common issue in orthopedic surgery, reported by multiple authors in the past for femoral head pin placements [10].

The current study was designed using a pragmatic setup simulating a real operative scaphoid fracture fixation while evaluating the effectiveness of common intraoperative fluoroscopic views in detecting articular screw penetration with open measurement through direct visualization as reference standard. Computed tomography was out of the scope of this study as it is neither commonly used during scaphoid fracture surgery, nor considered a reference standard for articular penetration (cartilage not being imaged).

This importance of the semi-pronated scaphoid view is consistent with the findings of Kim et al. that proximal screw penetration was best visualized on a 60° pronated oblique view [5]. However, the 45° pronated oblique view was not tested separately in their study so we cannot confirm that this one view is equally effective for both forms of penetration. In our study, pronating the scaphoid 15° further past the true semi-(45°)pronation slightly reduced the sensitivity of detecting STT screw penetration. This practical 45° view was studied for its higher intraoperative feasibility in daily practice, i.e., in the operative setting without a goniometer it is easier to estimate the angle halfway between 0° and 90° of pronation. For the same pragmatic reason, no combination of deviation and pronosupination was used, as reliability and feasibility of such views may be limited.

Consistent with their findings, small amounts of screw penetration (0.5 mm) were not always detected on fluoroscopic views as in two of out ten cases it remained unidentified on all views, and with 1 mm of penetration in one out of ten cases. Larger amounts of distal articular screw penetration 1.5–2 mm were detected in all cases with at least the 45° pronated oblique view. In contrast to their study assessing proximal articular screw penetration, no Steinmann pins were used in the present study. The choice for a standardized 24 mm cannulated screw was based on the aim to simulate a real surgical screw fixation as closely as possible.

One way to improve distal screw visualization on a true lateral wrist view is to radially deviation the wrist thereby flexing the scaphoid that displaces the screw head volarly and away from the trapezium. The 45° pronated oblique view was the most sensitive view for this purpose, detecting all of the up to 1.5 mm penetrating screws as well as providing the longest radiographically measurable screw penetration.

Another potential solution has been advocated by Patel et al. to use shorter screws as in their biomechanical study in 18 cadaveric scaphoids, in which maximizing screw length did not provide superior fixation [11]. They found no significant difference in ultimate failure load of a simulated scaphoid fracture by an oblique osteotomy between an 18 mm and 24 mm screw. However, their suggestion for shorter screws will not resolve the problem of insufficient screw advancement leading to screw head prominence.

The findings of the present study should be interpreted in light of its limitations inherent to a cadaveric setting, which may not perfectly reflect a clinical setting in the operating room, although in our experience it was an optimal simulation of reality. First, we focused only on STT screw penetration as it seems to be the most common contemporary problem related to hardware, and the fact that data on proximal screw penetration have not yet been published. Second, radiographic penetration past the outer cortical line likely overestimates true articular penetration as scaphoid cartilage thickness is estimated to be up to 1 mm in cadaveric wrists on the distal tubercle at the STT joint. This could in part explain the fact that on the 45° pronated oblique view the average protrusion was larger by 0.1 mm than measured by caliper, although the inherent imprecision to the latter methodology as well as differentiating increments of 0.5 mm should be taken in account. Alternative advanced imaging techniques would also have had inherent limitations such as the difficulty of discerning cartilage on CT and metal-induced artifacts on magnetic resonance imaging (MRI). Third, the combination of pronation and radial or ulnar deviation is difficult in specimens, but this is a variable that would be interesting to study for clinical application.

Even in case of minimal and clinically nonvisible screw penetration (Fig. 1B) of less than a millimeter, radiographic evaluation does not overestimate the loss of potential compression and stabilization forces of the screw as any screw thread purchasing merely the cartilage has significantly less holding strength compared with cortical bone. So the problem of protruding screws is twofold: less compression and stabilization, with a higher risk of symptomatology, secondary screw removal, and STT arthritis due to attrition of the screw in the long term [2, 4, 6]. This emphasizes its caution during surgery as it is an easily preventable problem.

In conclusion, standard anteroposterior and lateral fluoroscopy views of a percutaneous cannulated screw fixation of a scaphoid waist fracture are insufficient to detect STT screw penetration. According to the current study, standard views would have missed half the amount of screw penetrations, which seems to reflect the high incidence of this problem in current practice. The most sensitive view was the 45° pronated oblique view, which detected STT screw penetration in all cases.

## Data Availability

All data are available from the first author on request.
